# Effect of positive airway pressure on cardiac troponins in patients with sleep‐disordered breathing: A meta‐analysis

**DOI:** 10.1002/clc.23817

**Published:** 2022-03-21

**Authors:** Li‐Hua Wu, Cai‐Xia Hong, Zhi‐Wei Zhao, Yan‐Fei Huang, Huo‐Yu Li, Hong‐Ling Cai, Zhi‐Sen Gao, Zhi Wu

**Affiliations:** ^1^ Department of Respiratory and Critical Care Medicine The First Hospital of Putian City Putian Fujian Province People's Republic of China; ^2^ Department of Gynecology Zhangzhou Affiliated Hospital of Fujian Medical University Zhangzhou Fujian Province People's Republic of China; ^3^ Department of Otolaryngology Zhangzhou Affiliated Hospital of Fujian Medical University Zhangzhou Fujian Province People's Republic of China; ^4^ Department of Respiratory and Critical Care Medicine Zhangzhou Affiliated Hospital of Fujian Medical University Zhangzhou Fujian Province People's Republic of China

**Keywords:** cardiac troponin, meta‐analysis, positive airway pressure, sleep‐disordered breathing

## Abstract

**Background:**

Cardiac troponins are highly sensitive and specific biomarkers for cardiac injury. Previous studies evaluating the effect of positive airway pressure (PAP) on cardiac troponins in patients with sleep‐disordered breathing (SDB) have yielded conflicting results. The meta‐analysis was performed to examine the effect of PAP on cardiac troponins in SDB patients.

**Methods:**

PubMed, Web of Science, and EMBASE before September 2021 on original English language studies were searched. The data on cardiac troponins in both baseline and post‐PAP treatment were extracted from all studies. The data on the change of cardiac troponins in both PAP and control group were extracted from randomized controlled trials. Standardized mean difference (SMD) was used to synthesize quantitative results.

**Results:**

A total of 11 studies were included. PAP treatment was not associated with a significant change in cardiac troponin T between the baseline and post‐PAP treatment (SMD = −0.163, 95% confidence interval [CI] = −0.652 to 0.326, *z* = 0.65, *p* = .514). The pooled estimate of SMD of cardiac troponin I between the pre‐ and post‐PAP treatment was 0.287, and the 95% CI was −0.586 to 1.160 (z = 0.64, *p* = .519). The pooled SMD of change of cardiac troponin T between the PAP group and control group was −0.473 (95% CI = −1.198 to 0.252, *z* = 1.28, *p* = .201).

**Conclusions:**

This meta‐analysis revealed that PAP treatment was not associated with any change of cardiac troponin in SDB patients.

## 1 INTRODUCTION

Sleep‐disordered breathing (SDB) is a chronic condition characterized by repeated episodes of complete cessation or reduction of airflow during sleep. The resulting apneas and hypopneas can lead to intermittent hypoxemia and arousals from sleep. SDB encompasses a spectrum of abnormalities, including Cheyne–Stokes respiration (CSR), central sleep apnea (CSA), and obstructive sleep apnea (OSA). SDB is highly prevalent^1^ and is associated with many forms of cardiovascular disease (CVD) including hypertension, ischemic heart disease, atrial fibrillation and heart failure.

Troponin is a protein complex found in skeletal and cardiac muscles. Cardiac troponins (cTnI and cTnT) have been shown to be highly sensitive and specific biomarkers for myocardial cell injury allowing for early detection of acute coronary syndromes.^2^ In addition to the diagnostic value, elevated cardiac troponins were demonstrated to have a close correlation with adverse cardiovascular outcome whether coronary artery disease is present or not.^3^ Highly sensitive assays have been introduced that detect levels nearly 10‐fold lower than those detectable with the standard assay. The increased levels of cardiac troponin have been observed in SDB patients in previous studies.^4^, ^5^

Positive airway pressure (PAP, such as continuous PAP [CPAP], adaptive servo‐ventilation [ASV], or bi‐level PAP [BiPAP]) is the most effective nonsurgical treatment choice for moderate‐severe SDB. Whether PAP treatment has an impact on cardiac troponins in patients with SDB remains unclear. The purpose of this meta‐analysis was to quantitatively examine the effect of PAP treatment on cardiac troponins in patients with SDB.

## 2 METHODS

This meta‐analysis was reported in accordance with the Preferred Reporting Items for Systematic Reviews and Meta‐analysis (PRISMA) Statement for the conduct of meta‐analyses of intervention studies.^6^

### 2.1 Search strategy

A systematic computerized search of Web of Science, PubMed, and Embase was carried out by two independent investigators for publications from January 1975 to September 2021. The search was performed with a combination of MeSH and free terms. The search terms were as follows: (SDB or sleep apnea) and (positive airway pressure or noninvasive ventilation or adaptive servo‐ventilation) and (troponin).

### 2.2 Inclusion/exclusion criteria

Studies were considered eligible if they met the criteria as follow: (1) the population was limited to adults (age: >18 years old) with newly diagnosed CSA, OSA, or CSR; (2) the intervention was an application of PAP; (3) cTNI or cTNT was measured before and after PAP; (4) only English language publications were eligible.

Abstracts, case reports, conference articles, editorials, letters, expert opinions, reviews, animal studies, or those with insufficient data were excluded. The study with the largest population was selected if multiple studies reported data based on the same patient group. The corresponding author was contacted if the data reported in the studies were not enough to perform the statistical analysis. A study was excluded after two no‐response attempts. Two assessors independently reviewed each study, and disagreements were resolved by consensus.

### 2.3 Data extraction

Two reviewers independently extracted the data by using a standardized form for each relevant study. Information including first author, year of publication, country of the study, patient inclusion criteria, sample size, study design, participant characteristics, mean daily PAP usage time, duration of PAP therapy, troponin category, baseline, and posttreatment cardiac troponins were abstracted. The quality of randomized controlled trials (RCTs) was evaluated by Jadad score.^7^ The total score is 7 points. High quality was defined as ≥4 points, and low quality as a Jadad score with ≤3 points.

### 2.4 Statistical analysis

The software used for statistical analyses were STATA software (Version 12.0; Stata Corporation). Heterogeneity across studies was assessed by the *Q* test and the *I* ^2^ test. Significant heterogeneity was presented if *p* < .10 or *I* ^2^ > 50%. The fixed or random‐effects model was used for nonheterogeneous or heterogeneous data, respectively. The standardized mean difference (SMD) and 95% confidence interval (CI) were used to measure the effect size of an outcome. Begg's correlation and Egger's regression were performed to assess the risk of publication bias. The statistical significance was set at a two‐sided *p* < .05. If the mean or standard deviation of cardiac troponins could not be directly obtained from studies, we estimated the mean and deviation from the sample size, median, range, or interquartile range.^8^, ^9^

## 3 RESULTS

### 3.1 Literature search

Our search strategy yielded a total of 93 papers initially. The titles and abstracts of all the studies were reviewed and 16 studies were judged to be potentially relevant. After reviewing full text, five studies were further excluded for the following reasons: one was non‐English article, one study reports overlapping data, three studies lack essential data. Figure S1 summarized the detailed searching results.

### 3.2 Characteristics of the included studies

A total of 11 studies with 12 cohorts were identified eligible for inclusion. Of them, seven studies assessed cardiac troponin T,^10^, ^11^, ^12^, ^13^, ^14^, ^15^, ^16^ four studies assessed cardiac troponin I.^17^, ^18^, ^19^, ^20^ Four studies were RCTs^17^ and the remaining studies were observational. One study separated participants into two groups based on whether coexisting CAD.^12^ ASV therapy was adopted in two studies.^15^, ^16^ The characteristics of the 11 included studies were summarized in Tables 1 and 2. Three RCTs assessed cardiac troponin T^11^, ^15^, ^16^ and the information was presented in Table S1.

**Table 1:** Characteristics of the included studies

Study	Year	Nation	Sample size/male	Inclusion criteria	Ventilation duration/night (h)	Therapy duration	PAP type	Troponin category	CVD/HF	Study design
Lui	2021	China	46/36	AHI ≥ 15	3.1 ± 2.5	2 M	CPAP	hs‐cTnI	No	RCT
Mostafavi	2019	Iran	58/38	AHI ≥ 15	NR	2 W	PAP	hs‐cTnI	No	Observational study
Zhang	2018	China	28/25	AHI ≥ 30	6.4 ± 3.8	3 M	CPAP	hs‐cTnT	No	Observational study
Chang	2017	Australia	28/25	AHI ≥ 25	4.51 ± 2.1	2 M	CPAP	hs‐cTnT	No	RCT
Strehmel A	2016	Germany	21/16	AHI > 15	NR	1 d	CPAP	hs‐cTnT	CVD	Observational study
Strehmel B	2016	Germany	20/17	AHI > 15	NR	1 d	CPAP	hs‐cTnT	No	Observational study
Sharafkhaneh	2015	USA	13/13	AHI ≥ 15	5.3 ± 0.35	6 M	CPAP	hs‐cTnT	NR	Observational study
Maeder	2015	Switzerland	12/NR	AHI ≥ 15	NR	1 d	CPAP	hs‐cTnI	No	Observational study
Barcelo	2014	Spain	95/95	AHI ≥ 10	≥ 4	12 M	CPAP	hs‐cTnT	No HF	Observational study
Yoshihisa	2013	Japan	18/16	AHI > 15	>4	6 M	ASV	hs‐cTnT	HF (LVEF > 50%)	RCT
Yoshihisa	2012	Japan	42/37	AHI ≥ 15	NR	1 d	ASV	hs‐cTnT	HF (LVEF < 50%)	RCT
Çifçi	2010	Turkey	30/20	AHI > 5	NR	10 M	CPAP	cTnI	No	Observational study

Abbreviations: AHI, apnea‐hypopnea index; ASV, adaptive servo‐ventilation; CPAP, continuous positive airway pressure; cTnT, cardiac troponin T; cTnI, cardiac troponin I; CVD, cardiovascular disease; d, day; h, hour; HF, heart failure; hs, highly sensitive; LVEF, left ventricular ejection fractions; M, month; NR, not reported; PAP, positive airway pressure; RCT, randomized controlled trial; W, week.

**Table 2:** Patients' characteristics of the trials included in the meta‐analysis

Study	Age	AHI	LowSO_2_	BMI	Pre‐PAP cardiac troponins	Post‐PAP cardiac troponins
Liu	52.5 ± 9	49.76 ± 30.23	73.5 ± 13.0	30.9 ± 5.1	5.6 ± 6.1 pg/ml	4.8 ± 5.0 pg/ml
Mostafavi	49.89 ± 12.76	NR	NR	32.04 ± 4.39	0.061 ± 0.007 ng/L	0.097 ± 0.035 ng/L
Zhang	NR	47.7 ± 15.5	68.9 ± 11.9	28.8 ± 3.0	8.4 ± 2.4 pg/ml	7.6 ± 2.1 pg/ml
Chang	48 ± 12	41.8 ± 22.2	80 ± 10.2	31.95 ± 4.22	5.24 ± 2.40 ng/L	5.05 ± 2.28 ng/L
Strehmel A	61 ± 11	53 ± 21	71 ± 12	35 ± 7	9 ± 8 ng/L	10 ± 8 ng/L
Strehmel B	54 ± 12	49 ± 20	71 ± 15	33 ± 6	7 ± 3 ng/L	7 ± 3 ng/L
Sharafkhaneh	59.7 ± 2	50 ± 6	77 ± 3	36.5 ± 1.8	0.012 ± 0.0085 µg/L	0.011 ± 0.0035 µg/L
Maeder	54 ± 12 (*n* = 65)	39 ± 20 (*n* = 65)	NR	31.8 ± 6.3 (*n* = 65)	2.18 ± 1.27 pg/ml	2.10 ± 1.54 pg/ml
Barcelo	51 ± 9 (*n* = 133)	56 ± 25 (*n* = 133)	82 ± 3 (*n* = 133)	31 ± 6(133)	7.3 ± 3.4 ng/L	10.1 ± 4.9 ng/L
Yoshihisa	64.4 ± 15.7	37.0 ± 14.1	77.9 ± 9.9	24.8 ± 3.6	0.029 ± 0.018 ng/ml	0.015 + 0.009 ng/ml
Yoshihisa	62.0 ± 11.8	39.0 ± 17.3	79.1 ± 8.9	25.4 ± 4.6	0.042 ± 0.034 ng/ml	0.026 ± 0.017 ng/ml
Çifçi	49.97 ± 10.3	55.9 ± 26.1	NR	33.9 ± 4.8	0.007 ± 0.036 ng/ml	0.003 ± 0.018 ng/ml

Abbreviations: AHI, apnea‐hypopnea index; BMI, body mass index; LowSO_2_, lowest O_2_ saturation; NR, not reported; PAP, positive airway pressure.

### 3.3 Results for cTnT

**Figure 1:**
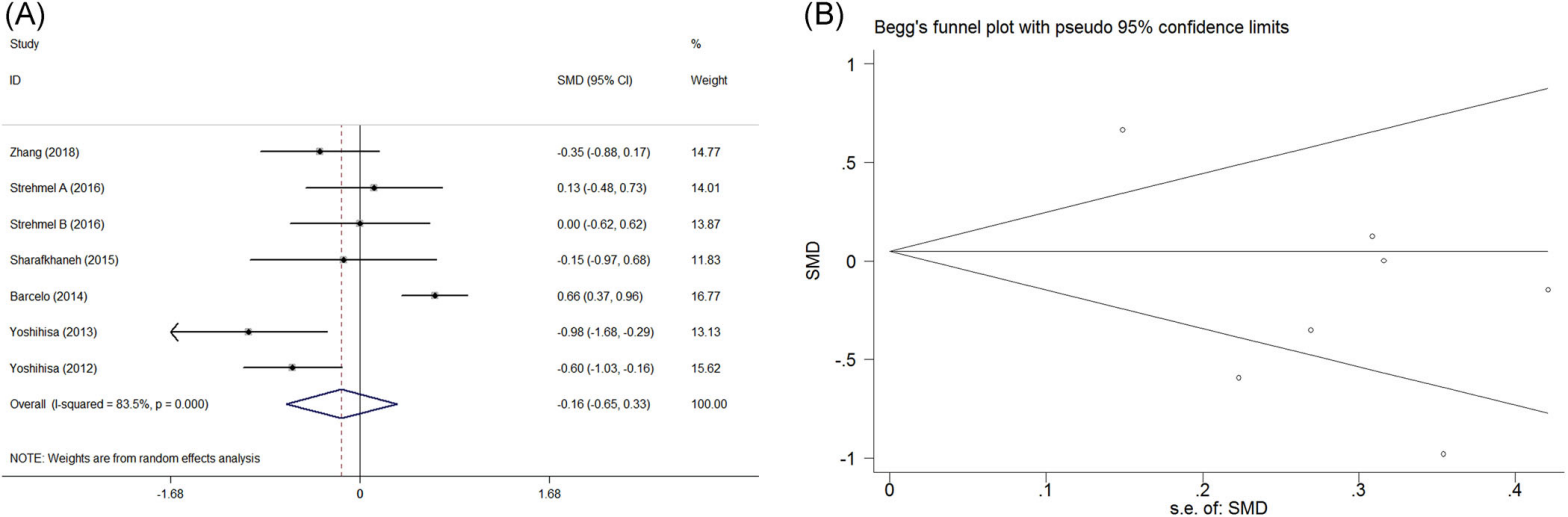
Forest plot for the change of cTnT before and after PAP treatment and funnel plots for assessing publication bias. (A) Forest plot for the change of cTnT before and after PAP treatment. (B) Funnel plots for assessing publication bias. cTnT, cardiac troponin T; PAP, positive airway pressure; SMD, standardized mean difference; SE, standard error

Seven studies evaluated the impact of PAP treatment on cTnT in SDB patients. As significant heterogeneity (*χ* ^2^ = 36.41, *I* ^2^ = 83.5%, *p *< .001) was noted, random‐effect model was performed. Pooling the data showed that PAP treatment was not associated with a significant change in cTnT between the baseline and post‐PAP treatment (SMD = −0.163, 95% CI = −0.652 to 0.326, *z* = 0.65, *p* = .514) (Figure 1A). No significance of publication bias existed in our meta‐analysis (Egger's test, *p* = .099; Begg's test, *p* = .764) (Figure 1B).

### 3.4 Results for cTnI

**Figure 2:**
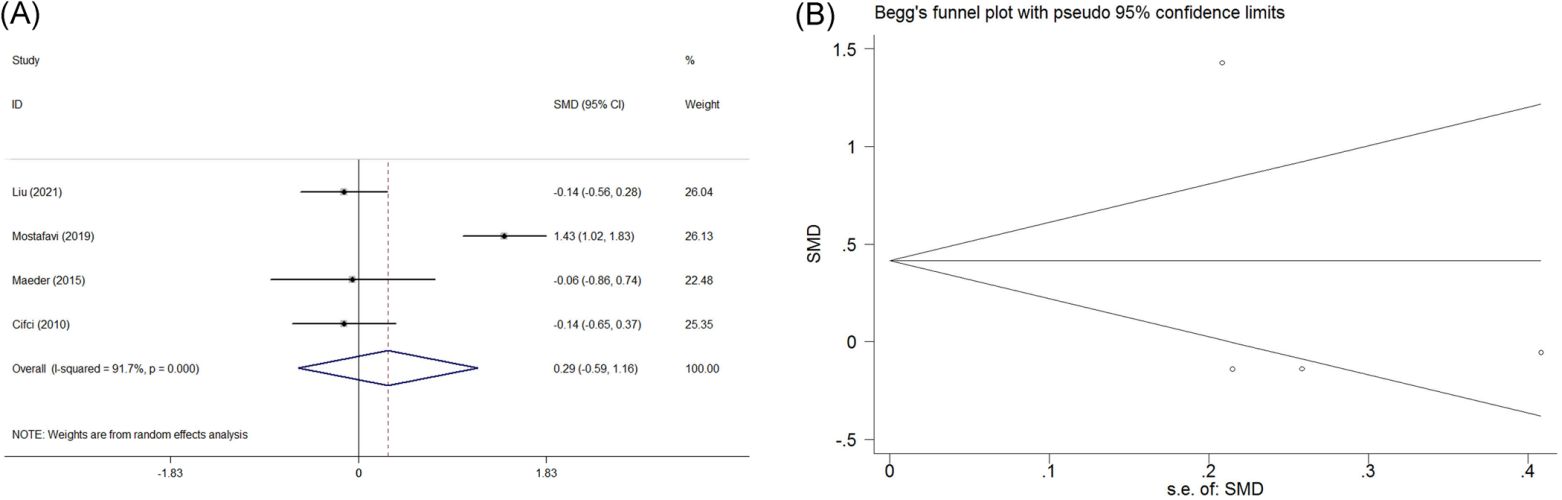
Forest plot for the change of cTnI before and after PAP treatment and funnel plots for assessing publication bias. (A) Forest plot for the change of cTnI before and after PAP treatment. (B) Funnel plots for assessing publication bias. cTnI, cardiac troponin I; PAP, positive airway pressure; SE, standard error; SMD, standardized mean difference

Four studies reported the data of cTnI before and after PAP treatment. The pooled estimate of SMD between the pre‐ and post‐PAP treatment groups was 0.287, and the 95% CI was −0.586 to 1.160 (*z* = 0.64, *p* = .519, random‐effect model) (Figure 2A). This indicated that PAP treatment has no impact on the change of cTnI. There was a significant heterogeneity across studies (*χ* ^2^ = 36.24, *I* ^2^ = 91.7%, *p* < .001). Begg's tests (*p* = 1.000) and Egger's tests (*p* = .615) indicated no evidence of publication bias in the present study (Figure 2B).

### 3.5 Results for RCTs

**Figure 3:**
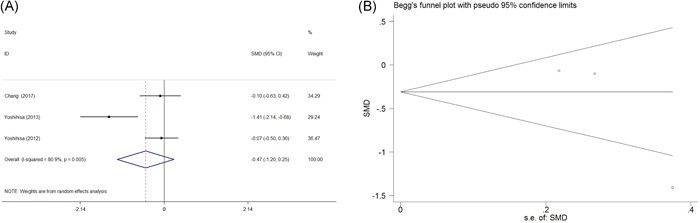
Forest plot for the change of cTnT between PAP treatment and control group in three RCTs and funnel plots for assessing publication bias. (A) Forest plot for the change of cTnT between PAP treatment and control group. (B) Funnel plots for assessing publication bias. cTnT, cardiac troponin T; PAP, positive airway pressure; SE, standard error; SMD, standardized mean difference; RCTs, randomized controlled trials

cTnT was evaluated in three RCTs. The analysis showed *χ* ^2^ = 10.45, *I* ^2^ = 80.9%, and *p* = .005, indicating that the studies were heterogeneous. A random‐effect model was adopted for the pooled analysis. The pooled SMD of change of cTnT between the PAP group and control group was −0.473 (95% CI = −1.198 to 0.252, *z* = 1.28, *p* = .201) (Figure 3A). There was no significance of publication bias in our meta‐analysis (Egger's test, *p* = .238; Begg's test, *p* = .296) (Figure 3B).

## 4 DISCUSSION

The present study including 11 studies (12 cohorts) aimed to examine the effect of PAP treatment on cardiac troponins in SDB patients. The results demonstrated that there was no significant change in cTnT or cTnI between pre‐ and post‐PAP treatment. The results of three RCTs also revealed no difference in change of cTnT between control and PAP treatment group. The findings revealed that PAP therapy exerted no impact on cardiac troponins in subjects with SDB.

Highly sensitive cardiac troponin has been recognized as a key marker of myocardial injury. It is widely used for the diagnosis, risk stratification, and prognostic assessment of ACS and other CVD. In a population‐based cohort, highly sensitive cTnT was found to be associated with structural heart disease and subsequent risk for all‐cause mortality.^21^ The results from another prospective observational cohort study also confirmed the above findings.^22^ A prospective study including 9698 participants without known coronary heart disease/stroke at baseline reported that hs‐cTnT was associated with incident coronary heart disease, heart failure, and mortality.^23^ Zhu et al.^24^ followed up Busselton Health Study Cohort and found that hs‐cTnI is an independent predictor of fatal and nonfatal CVD events.

The relationship between SDB and cardiac troponins has been explored for many years. In 2004, a prospective cohort study demonstrated that OSA patients with coexisting CAD do not have nocturnal episodes of myocyte necrosis evidenced by detectable serum cTnT.^25^ However, a standard assay but not highly sensitive assay was adopted in this study. A community‐based study including 1645 participants free of heart failure and coronary heart disease reported that the severity of OSA was significantly correlated with higher hs‐TnT levels after adjusting for potential confounders. After a median of 12.4 years follow‐up, hs‐TnT was found to be related to incident heart failure or risk of death in OSA patients.^4^ Einvik et al.^5^ analyzed cTnI with high sensitivity troponin assay and demonstrated that increased more severe OSA is independently related with higher concentrations of hs‐TnI. These indicated that SDB might cause low‐grade myocardial injury.

PAP therapy has been demonstrated to improve hypertension,^26^ cardiac systolic and diastolic function,^27^, ^28^ arterial stiffness,^29^ and vascular endothelial function^30^ in SDB patients. These are all risk factors of cardiac injury. So it would therefore be logical to assume that PAP treatment would also result in lower levels of cardiac troponin. However, our study failed to show the benefit of PAP treatment in lowering the levels of cTnT or cTnI. We speculated that several reasons might possibly explain this result. First, most of subjects included were free of CVD or heart failure. Subjects with CVD or heart failure were potentially susceptible to the hypoxemic effects of OSA, thus, PAP treatment might be more effective in this population group. Second, the baseline value of cardiac troponins in most of studies was not higher than normal range, which limited the extent of change induced by PAP treatment. Third, highly sensitive cardiac troponins are sensitive to cardiac damage or necrosis rather than ischemia or hypoxia. The negative results suggested that cardiac troponins were insufficient to predict cardiac effects of CPAP therapy in SDB patients without CVD.

Our study has several strengths. To our knowledge, this was the first meta‐analysis to examine the effect of PAP treatment on cardiac troponins in subjects with SDB. In addition, the results of pool‐analysis of three RCTs which compared the change of cTnT between PAP group and control group were consistent with those of pool‐analysis of seven studies which compared the change of cTnT before and after PAP treatment. Furthermore, neither cTnT nor cTnI was influenced by PAP treatment in SDB patients. However, several limitations were to be noted. A limitation of the study was small sample size and limited number of studies. Another limitation of meta‐analysis was that the heterogeneity was present among individual studies. Finally, SMD was chosen as effect estimates as both cTnT and cTnI were measured and expressed differently.

In our meta‐analysis, PAP treatment was not associated with any change of cardiac troponin in SDB patients. The present findings are inconclusive, further prospective large‐scale studies with large sample size are required to explore PAP treatment effect on cardiac troponin in some specific patient group.

## CONFLICTS OF INTEREST

The authors declare no conflicts of interest.

## Supporting information

Supplementary information.

Supplementary information.

## Data Availability

The data that support the findings of this study are available from the corresponding author upon reasonable request.
