# Supplementary figures and images for: Effect of positive airway pressure on cardiac troponins in patients with sleep‐disordered breathing: A meta‐analysis
Source: Clin Cardiol. 2022 Mar 21;45(5):567–73. doi: 10.1002/clc.23817 (PMC9045066; doi:10.1002/clc.23817)

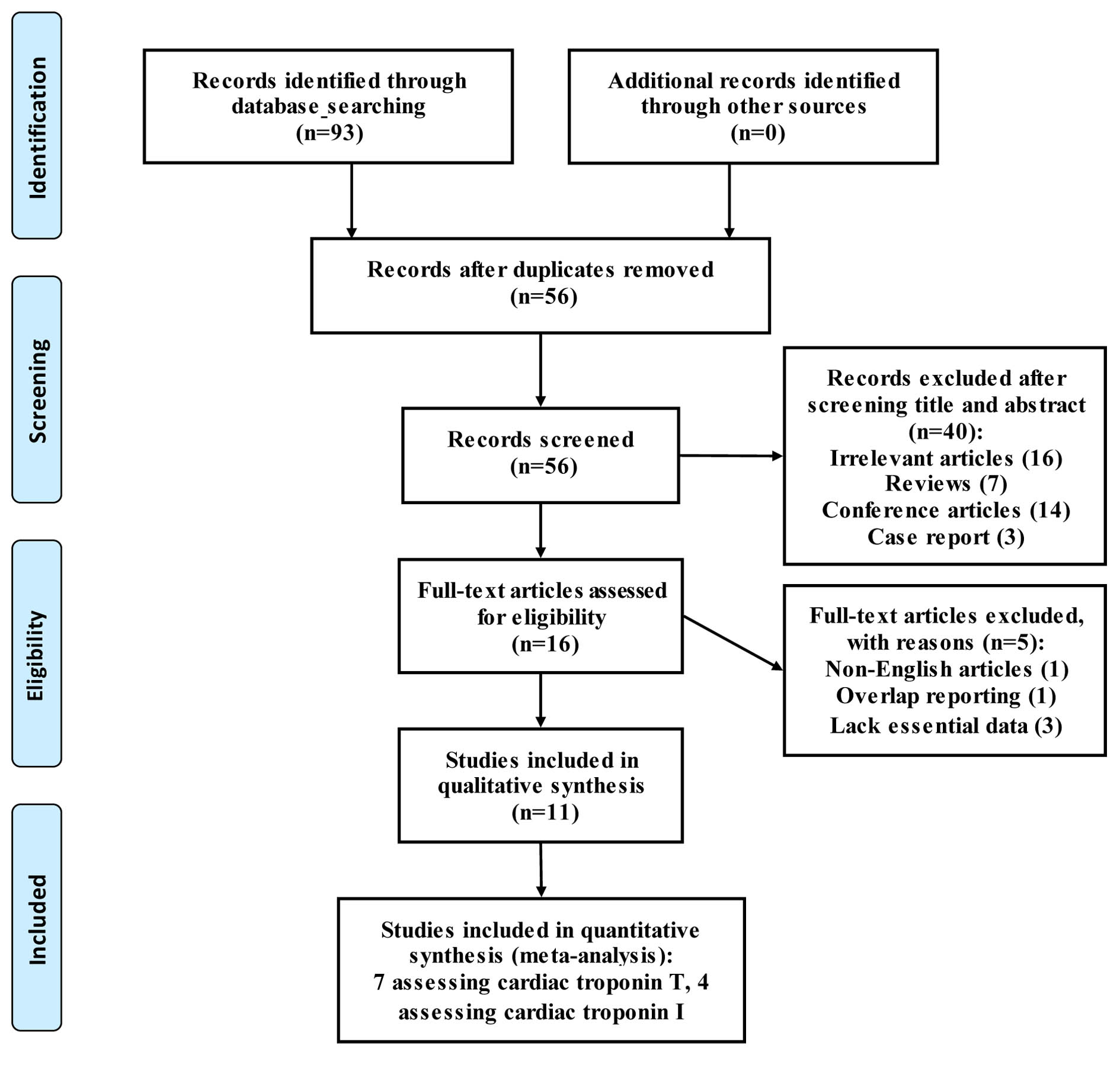

Supplement: Supplementary file 1 — Supplementary information. [file CLC-45-567-s002.jpg]
