# Supplementary material for: Effect of positive airway pressure on cardiac troponins in patients with sleep‐disordered breathing: A meta‐analysis
Source: Clin Cardiol. 2022 Mar 21;45(5):567–73. doi: 10.1002/clc.23817 (PMC9045066; doi:10.1002/clc.23817)
Supplement: Supplementary file 2 — Supplementary information. [file CLC-45-567-s001.docx]

Table S1 Characteristics of 3 RCTs

| Author | Year | Study design | Treatment | Sample size | Age | AHI | LowSO_2_ | BMI | Pre-PAP cTnT | Post-PAP cTnT | Jadad score |
| --- | --- | --- | --- | --- | --- | --- | --- | --- | --- | --- | --- |
| Chang (treatment group) | 2017 | Crossover | CPAP | 28 | 48±12 | 41.8±22.2 | 80±10.2 | 31.95±4.22 | 5.24±2.40 ng/L | 5.05 ± 2.28 ng/L | 4 |
| Chang (control group) | 2017 | Crossover | Sham  CPAP | 28 | 48±12 | 41.8±22.2 | 80±10.2 | 31.95±4.22 | 5.24±2.40 ng/L | 5.29 ± 2.28 ng/L | 4 |
| Yoshihisa (treatment group) | 2013 | Parallel | ASV | 18 | 64.4±15.7 | 37.0±14.1 | 77.9±9.9 | 24.8± 3.6 | 0.029±0.018 ng/mL | 0.015+0.009 ng/mL | 1 |
| Yoshihisa B(control group) | 2013 | Parallel | Non-ASV | 18 | 64.3+12.4 | 36.0+15.7 | 77.9+10.3 | 25.2+3.1 | 0.053+0.027 ng/mL | 0.068+0.031 ng/mL | 1 |
| Yoshihisa (treatment group) | 2012 | Crossover | ASV | 42 | 62.0±11.8 | 39.0±17.3 | 79.1±8.9 | 25.4±4.6 | 0.042±0.034 ng/ml | 0.026±0.017 ng/ml | 1 |
| Yoshihisa (control group) | 2012 | Crossover | Oxygen | 42 | 62.0±11.8 | 39.0±17.3 | 79.1±8.9 | 25.4±4.6 | 0.042±0.034 ng/ml | 0.028±0.018 ng/ml | 1 |

Abbreviation: RCTs=randomized controlled trials, CPAP=continuous positive airway pressure, ASV=adaptive servo-ventilation, PAP=positive airway pressure, cTnT=cardiac troponin T, AHI=apnea-hypopnea index, LowSO_2_=lowest O_2_ saturation, BMI=body mass index.
